# The Body of Evidence: What Can Neuroscience Tell Us about Embodied Semantics?

**DOI:** 10.3389/fpsyg.2013.00050

**Published:** 2013-02-13

**Authors:** Olaf Hauk, Nadja Tschentscher

**Affiliations:** ^1^MRC Cognition and Brain Sciences UnitCambridge, UK

**Keywords:** embodiment, semantics, neuroimaging, fMRI, EEG/MEG, TMS

## Abstract

Semantic knowledge is based on the way we perceive and interact with the world. However, the jury is still out on the question: to what degree are neuronal systems that subserve acquisition of semantic knowledge, such as sensory-motor networks, involved in its representation and processing? We will begin with a critical evaluation of the main behavioral and neuroimaging methods with respect to their capability to define the functional roles of specific brain areas. Any behavioral or neuroscientific measure is a conflation of representations and processes. Hence, a combination of behavioral and neurophysiological interactions as well as time-course information is required to define the functional roles of brain areas. This will guide our review of the empirical literature. Most research in this area has been done on semantics of concrete words, where clear theoretical frameworks for an involvement of sensory-motor systems in semantics exist. Most of this evidence still stems from correlational studies that are ambiguous with respect to the behavioral relevance of effects. Evidence for causal effects of sensory-motor systems on semantic processes is still scarce but evolving. Relatively few neuroscientific studies so far have investigated the embodiment of abstract semantics for words, numbers, and arithmetic facts. Here, some correlational evidence exists, but data on causality are mostly absent. We conclude that neuroimaging data, just as behavioral data, have so far not disentangled the fundamental link between process and representation. Future studies should therefore put more emphasis on the effects of task and context on semantic processing. Strong conclusions can only be drawn from a combination of methods that provide time-course information, determine the connectivity among poly- or amodal and sensory-motor areas, link behavioral with neuroimaging measures, and allow causal inferences. We will conclude with suggestions on how this could be accomplished in future research.

## Introduction

It seems obvious that the way we interact with the world shapes the way we represent concepts and knowledge. However, the degree to which experience shapes concepts and cognitive strategies in the fully developed brain is still poorly understood. In its most general form, theories of embodied cognition assume that human mental functions are shaped by the way the human body interacts with the environment (Varela et al., 1992; Clark, 1997; Barsalou, 2008). Theories differ with respect to the degree of embodiment they consider relevant, and assumptions range from that the environment is part of cognition, that the goal of perception is action, and that sensory-motor systems aid cognitive processes (e.g., Wilson, 2002). In the neuroscience of semantics, the debate focuses mainly on the question as to what degree perceptual and motor systems of the brain contribute to semantic representations and processes (Barsalou et al., 2003; Fischer and Zwaan, 2008; Nazir et al., 2008; Knoeferle et al., 2010; Kiefer and Pulvermüller, 2011; Pulvermüller, 2012). Even in this relatively circumscribed research field, the views on the relevance of embodiment range from “strongly embodied” to “fully disembodied” (Meteyard et al., 2010). It therefore seems important to ask what we mean by embodiment in a specific context, and what type of evidence we accept to determine its relevance. It is unlikely that there is a one-fits-all definition of embodiment, and we may find that sensory-motor systems contribute a lot to one aspect of cognition (e.g., semantics or mental imagery), but hardly at all to another (e.g., arithmetic problem solving).

Our main aim for this article was to formulate the major methodological challenges for neuroscientific research on embodiment, and offer suggestions on how different methods can be used to answer specific questions of embodied semantics. How can we test theories of embodied semantics? Which methods are suitable to test what type of predictions? We are not attempting to develop another theory of semantics, but rather ask what type of questions can be answered with existing methodology. On this basis, we will provide a review of studies dealing with embodied semantics for single-words and numerical cognition. These are well-focused research areas, in which a large body of behavioral and neuroscientific evidence has already been acquired. They are therefore well-suited to illustrate the methodological and theoretical challenges of the research area, and it should be expected to be the most likely candidates to converge on a general conclusion.

## Motivation for Embodied Theories of Semantics

Few papers on the neuroscience of embodied semantics contain an explicit definition of “(semantic) representation”. It is implicitly assumed that it refers to an implemented code or symbol system that stands for external entities. Marr (1982) provides the definition “A representation is a formal system for making explicit certain entities or types of information, together with a specification of how the system does this” (chap. 1.2). It is not obvious how this relates to measures of brain activation, in particular with respect to the second part of the definition.

In this section, we will briefly summarize the main theoretical motivations for investigations on embodied semantics. The starting point for most embodied approaches is the question: how can a network of symbolic relationships relate to the real world? At some point, the tree of symbolic relationships should be grounded in sensory-motor experience. This is the “symbol grounding problem” of artificial intelligence (Harnad, 1990): one can write sophisticated computer programs that transform and process symbols that stand for semantic representations, but it is not clear how these symbols acquire meaning or intentionality. This problem is illustrated in Searle (1980)’s “Chinese room” problem: an English speaker who manually executes an algorithm to translate written English symbols into written Chinese symbols does not necessarily “understand” Chinese herself. Harnad suggests a “hybrid” symbolic/non-symbolic model, in which abstract functions can emerge by means of “bottom-up grounding” of categories from grounded sensory representations. Similarly, Barsalou’s theory of perceptual symbol systems suggests that conceptual knowledge is represented as bottom-up and top-down interactions between sensory-motor systems and higher-level association areas (Barsalou, 1999). These higher-order cortices, convergence zones, or convergence regions have been localized to different parts of the brain (Damasio et al., 2004; Binder and Desai, 2011).

Mechanistic models have been proposed to explain how sensory-motor areas of the brain become connected with core language areas based on Hebbian principles of association learning (Hebb et al., 1971; Braitenberg and Pulvermüller, 1992; Pulvermüller, 1999; Wennekers et al., 2006). However, this does not necessarily imply that the fully developed brain cannot represent information independently of its original source. In order to categorize a word or an object as a horse, we may not have to invoke a full picture of a horse, but instead this decision may be made in higher-level association areas alone. Taken to the extreme, embodied theories of semantics might suggest that we need to activate the retina in order to understand the word “rose” – but if we consider this implausible, then we cannot argue for activation in visual cortex on purely theoretical grounds either. In the interest of speed and accuracy, it may even be more optimal to represent some information in local and specialized rather than distributed brain networks. Obviously, solid empirical evidence is needed as to whether or when sensory-motor systems contribute to semantic processes.

A particularly challenging case for theories of embodiment are abstract words, since they have no obvious referents in sensory-motor experience. Possible approaches to the incorporation of abstract semantics in frameworks of embodiments are for example summarized by Glenberg et al. (2008) and Pecher et al. (2011). First, abstract semantics can rely on concrete concepts by means of metaphor or image schemas (Lakoff, 1987; Gibbs and Steen, 1999). For example, the abstract knowledge that a proposition can be true or false, but not both, may be based on the sensory experience that an object can be either inside or outside a container, but not both. Second, some abstract concepts can be based on generalizations from situated simulations (Barsalou, 1999). For example, the concept of “truth” may be based on repeated experience of the consistency between simulated predictions (e.g., in order to verify a statement such as “the cup is on the table”) and perception (seeing the cup on the table). Third, Glenberg and Robertson (1999) formulated the “indexical hypothesis”, which states that abstract propositions can be acquired on the basis of concrete concepts. For example, abstract information transfer (such as reading something to someone) is grounded in concepts that describe the transfer of objects. It has also been found that not only sensory-motor, but also affective-emotional experience, shapes the processing of abstract words (Kousta et al., 2011).

As has been pointed out before (Kiefer and Pulvermüller, 2011), the embodiment of abstract semantics has only been addressed by a few neuroscientific studies yet. However, questions about the role of sensory-motor systems have also been asked in the context of numerical cognition (Lakoff and Nunez, 2000). For example, the “mental number line” has been suggested as the basis of the “SNARC” effect (Dehaene et al., 1993; Wood et al., 2008), and effects of finger-counting habits on number processing have recently been reported (Tschentscher et al., 2011; Fischer et al., 2012). We therefore included number processing in our review on embodied abstract semantics.

## The Mental Imagery Debate

Some theoretical and methodological issues regarding the involvement of sensory-motor processes in higher cognition have already been raised in the debate about the role of pictorial representations in mental imagery, which started about four decades ago before the appearance of modern neuroimaging (e.g., Paivio, 1971; Kosslyn, 1975). The most relevant part of this debate for the present review is the question put forward by Anderson (1978)^1^: can our experiments actually distinguish between different types of representations (e.g., visual or propositional, abstract or modality-specific)? Similar questions have been asked by other authors more recently (Thomas, 1999; Pylyshyn, 2002), but in this section we will follow the line of argument presented by Anderson (1978).

Anderson formally decomposed the information processing sequence between stimulus perception and response execution using operators: an operator *E* for stimulus encoding, which turns a stimulus *S*_i_ into an internal representation *I*_j_ (i and j do not have to be the same). There can be internal transformations *T* that can transform representations into different formats, such that *T*(*I*_i_) = *I*_j_. Finally, there are decoding operators that associate responses *R* with representations, i.e., *R*_j_ = *D*(*I*_i_). The basis of the argument is that “If we restrict ourselves to behavioral data, we cannot directly observe the internal processes *E*, *T*, and *D* nor the internal representations” (p. 263). What we measure is necessarily a conflation of encoding, transformation, and decoding processes. Thus, one model with specific assumptions about the structure of internal representations can be mimicked by another model with different assumptions about internal representations, when appropriate choices for the other operations are made. In other words, what we measure reflects a representation-process pair, and a change in assumptions about representations can be compensated for by changes in assumptions about processes. Anderson admits that there may be further constraints on representations and processes, e.g., based on parsimony, although he calls parsimony “an unfortunately subjective concept” (p. 266). However, he demonstrates – e.g., in a detailed analysis of letter rotation – that in the field of mental imagery, the most important behavioral evidence can be accommodated by both pictorial and propositional accounts.

Anderson offers an intriguing solution to this dilemma: just live with it. The fact that another theory also explains the data does not mean that either theory is useless (e.g., wave or particle theories of light are still useful in different contexts, and a general theory linking the two took a long time to develop). But even if we accept the argument that two competing theories can be useful, we of course still strive to find the most general theory that may comprise both as special cases. In order to do so, the only possibility is to find new sources of evidence.

Neurophysiological methods are a promising possibility, since they may bring us closer to “online” processing in the brain. If any methodology would allow us to measure a representation or an operation directly, we would be one step ahead. However, as Anderson notes, there are serious reasons for challenging neurophysiological data because “they do not provide anything like direct observation of the mental objects” (p. 271). Similar arguments have been put forward by other authors (Page, 2006). In principle, the logic about the conflation of representations and processes also applies to neurophysiological measurements. Certain parsimony and plausibility constraints may be justified – but they *have* to be justified. We therefore need to understand what we measure, and how we can relate this to our models and theories. This will be discussed in the following section.

## Neuroscientific Methods for the Investigation of Semantics

In the previous section, we highlighted the limitations of behavioral data for revealing semantic representations. Here, we will introduce the most common neuroimaging methods that have been employed in this endeavor. Neuroimaging data clearly exceed behavioral data in complexity. The hope is that the information contained in spatio-temporal patterns of brain dynamics allows specific conclusions about perceptual or cognitive processes and representations (e.g., Henson, 2005). However, three major problems complicate the interpretation of neuroimaging data:

1) Activation can be ambiguous with respect to the processing stage at which it occurs;
2) Activation is correlational, and may be epiphenomenal and not causally related to the processes of interest;
3) There is no one-to-one relationship between brain areas and cognitive functions.

Problem 1 is particularly important for metabolic neuroimaging, where the measured entity (e.g., the hemodynamic BOLD response) has a temporal resolution of several seconds or more (e.g., Buckner, 1998). Activation observed with these methods may occur at the processing stage of interest, or at any later stage that is sensitive to its output. For example, if the semantic decoding of the word “hammer” leads to the activation of mental imagery processes or episodic memories involving a hammer, then the latter may cause activation in motor cortex, not the former. In order to interpret fMRI contrasts, whether in a univariate or multivariate manner, one needs to account for processes up to several seconds after stimulus onset. This can be a challenging task when contrasting stimuli that can easily be categorized by participants, such as words vs. line drawings, words vs. pseudowords, or for different semantic word categories.

This problem can be addressed using methods with high temporal resolution in the millisecond range (such as EEG/MEG), which may distinguish “early” processes (e.g., lexico-semantic information retrieval) from “late” ones (such as mental imagery). One can plausibly argue that activation that occurs in latency ranges of earliest lexico-semantic information retrieval (e.g., around 200 ms after stimulus onset) is too early to reflect mental imagery processes (Pulvermüller, 1999; Hauk and Pulvermüller, 2004).

However, problem 2 still remains: activation may be triggered by a stimulus in a spreading-activation, automatic association, or conditioned manner, but it may not contribute causally to the process of interest. As Mahon and Caramazza (2008) have pointed out, there may be at least four possible explanations for early activation in sensory-motor areas, e.g., in response to the word “kick”: “(1) the word ‘kick’ directly activates the motor system, with no intervening access to abstract conceptual content; (2) the word ‘kick’ directly activates the motor system and in parallel activates abstract conceptual content; (3) the word ‘kick’ directly activates the motor system and then subsequently activates an abstract conceptual representation; and finally, (4) the word ‘kick’ activates an abstract conceptual representation and then activates the motor system.” As an illustration, these authors point to the example of the Pavlovian dog: the fact that it salivates as soon as it hears the bell does not mean that salivating contributes to the recognition of the sound of the bell. Effects occurring in the same latency range (e.g., sensory-motor activation in a latency range associated with lexico-semantic access) do not necessarily imply that the underlying processes affect each other.

In order to show that activation in a brain region causally affects a process, one has to reverse the logic of neuroimaging: instead of measuring brain activity in response to a stimulus or task, one needs to modulate activity in a brain region and measure the effect on performance. A non-invasive technique that allows short term stimulation of specific brain areas with reasonable spatial resolution is transcranial magnetic stimulation (TMS; Pascual-Leone et al., 2000). Short pulses of magnetic fields, usually applied via palm-sized coils, induce electrical currents in the underlying brain tissue. These pulses can induce muscle twitches in thumbs or legs, or lead to temporary speech arrest (e.g., Devlin and Watkins, 2006). Stimulation can lead to “temporary lesions,” i.e., impair the function of a brain area, which can be reflected in slower response times or higher error rates. Conversely, it is also possible to “prime” a brain area, which can result in improved performance. The physiological mechanisms that lead to either of these outcomes are not yet fully understood. The effect of stimulation of particular brain areas on behavioral performance provides the strongest non-invasive evidence that these brain areas indeed contribute to the process of interest. A potential problem is that induced activation may spread from one brain area to other connected ones. This can potentially be a problem when we want to distinguish effects in primary motor cortex from premotor cortex, or primary sensory areas from higher-level areas. A possible alternative to TMS is transcranial direct current stimulation (tDCS; Paulus, 2011). However, it is not as spatially specific as TMS (at least with current technology).

We would like to point out that studies using motor-evoked potentials (MEPs; e.g., Buccino et al., 2005; Papeo et al., 2009), which show a modulation of excitability of motor areas during language comprehension, do not demonstrate a causal role of motor cortex in language comprehension. Just as EEG/MEG and fMRI, they only show that language processing affects motor cortex, but not vice versa. We will therefore focus on studies that have studied the effect of motor cortex stimulation on language performance.

Another possibility to investigate the effect of “modulation” of brain activity on behavior is to study patients with specific brain impairments, e.g., after stroke. The number of studies that can be run in this way is obviously limited. It can be difficult to establish the spatial specificity of brain lesions, their knock-on effects on connected brain areas, and the effects of neuronal plasticity. Furthermore, severe damage to general language or semantic functions may mask more subtle effects, e.g., for different word categories. This may be the reason why Binder and Desai (2011) recently concluded that “conceptual deficits in patients with sensory-motor impairments, when present, tend to be subtle rather than catastrophic” (p. 531). We included neuropsychological studies in our review where we felt appropriate, but a detailed discussion of the limitations of this methodology is not within the scope of this paper.

The demonstration that a brain area is activated in a particular contrast, and that activation in this brain area predicts performance, still does not uniquely define the function of this area (point 3 above). The same brain area could, in principle, serve different functions depending on which neurons are active or which other brain areas are involved. All available non-invasive neuroimaging methods rely on signals produced by large numbers (thousands to millions) of neurons and synapses. Furthermore, brain areas are highly interconnected. The finding that an area is active during finger movement as well as during language comprehension does not directly imply that exactly the same processes of the former are involved in the latter. Experimentally, priming or repetition suppression paradigms can potentially address the problem whether the same or different neuronal populations are involved in different processes (Henson and Rugg, 2003): if two processes involve different neurons in the same area, they may not prime each other.

We conclude that there is no single brain measure that can be interpreted in terms of “representations” or unique processes in a straight forward manner. For word processing, this means that the activation of sensory-motor areas is consistent with their involvement in semantics, but it is not a proof. TMS studies have the potential to provide crucial evidence for the causal involvement of a brain area in cognitive processes, but it may still not uniquely determine the functional role of the targeted brain areas, which requires further experimental manipulations.

## Review of the Empirical Literature

In this section, we will test how the methodological constraints formulated above have been addressed in the empirical literature. We will begin with behavioral evidence for the view that sensory-motor knowledge affects language processing, followed by evidence from metabolic neuroimaging (mostly fMRI) studies for activation of sensory-motor systems during semantic processing, and by an analysis of the time-course of these effects based on EEG/MEG studies. Finally, we will ask whether there is evidence for a causal role of sensory-motor systems in semantics, mostly relying on TMS data.

### Concrete words

#### Behavior

Behavioral evidence for an interaction between action and language was provided for example by the Action-Sentence-Compatibility-Effect (ACE): participants performed a hand movement faster when the direction of movement was congruent with the direction of movement described in a preceding sentence, compared to when it was incongruent (Glenberg and Kaschak, 2002). Similar interference effects have been observed with visual and auditory motion paradigms (Kaschak et al., 2005, 2006). Evidence for this type of interaction has also been provided for single-word processing. When participants were required to perform a grasping movement triggered by the visual presentation of a word, movement kinematics changed depending on whether the word was hand-action-related or not (Boulenger et al., 2006). Effects of action-word type on response execution have also been documented in other studies (Dalla Volta et al., 2009; Mirabella et al., 2012). Meteyard et al. (2007) demonstrated that listening to verbs that described an upward or downward motion interfered with performance in a motion detection task, i.e., perceptual sensitivity was impaired when verb motion and displayed motion were incongruent. Similarly, motion direction of dot patterns and indicated by visually presented words interfered in a lexical decision task (Meteyard et al., 2008). The fact that these interference effects occurred when motion patterns were presented near the perceptual threshold, rather than supra-threshold, was taken as evidence that “automatic activation of motion-responsive area MT+ … gives rise to the interference between perceptual and semantic information processing” (p. R732). These results demonstrate that interference between semantics and perceptual-motor information occurs at the behavioral level. However, it is not direct evidence that this interference is due to an overlap of semantic and perceptual-motor systems. The interference could still occur at a separate level which is sensitive to the congruency of output among different processes.

#### fMRI and PET

A number of fMRI and PET studies have shown that sensory-motor areas become active during language comprehension, mostly in the action domain (Hauk et al., 2004; Tettamanti et al., 2005; Aziz-Zadeh et al., 2006; Kemmerer et al., 2008; Boulenger et al., 2009; Raposo et al., 2009; Willems et al., 2010b; Hauk and Pulvermüller, 2011; Pulvermüller et al., 2011), but also for auditory (Kiefer et al., 2008), visual (Pulvermüller and Hauk, 2006; Simmons et al., 2007; Hauk et al., 2008a), and olfactory-gustatory concepts (Gonzalez et al., 2006; Barros-Loscertales et al., 2012), and for a mixture of those (Noppeney and Price, 2002, 2003; Hauk et al., 2008a; Kiefer et al., 2012).

The finding of category-specific differences in sensory-motor areas is usually directly taken as evidence that neuronal representations of semantic knowledge have been shaped by individual experience. An interesting test case are left- and right-handers: does the way we usually perform an action shape the way we represent is semantically? Two studies on this issue have led to different results. Willems et al. (2010a) reported more activation in left motor cortex when right-handers read uni-manual action-related words (e.g., “throw”), while left-handers showed more activation in right motor areas. Hauk and Pulvermüller (2011) used uni-manual and bi-manual (e.g., “clap”) words, and found that uni-manual words activated left motor cortex in both left- and right-handers, while bi-manual words activated motor cortex bilaterally in both groups. The authors of both studies interpreted their results in terms of embodied semantics. However, while the former argued that “implicit mental simulation during language processing is body specific” (abstract), the latter concluded that their results reflect the influence of “left-hemispheric language dominance on the formation of semantic brain circuits on the basis of Hebbian correlation learning” (abstract). These conclusions are not contradictory. Differences between these studies may be explained by the use of different tasks. Willems et al. (2010a) used a lexical decision task, which engages motor areas and may focus participants’ attention to action-related aspects of the stimuli. Hauk and Pulvermüller used a silent reading task that did not require an explicit response. This should stimulate further research into the effects of task demands on brain activation during semantic processing.

Some of the previous empirical results have been questioned on empirical grounds. For example, Postle et al. (2008) did not find somatotopic activation to action-words in their fMRI study, and pointed out that evidence for motor cortex activation to action-words in cytoarchitectonically defined motor areas is inconsistent across studies. Nevertheless, taken together these studies provide strong evidence for the differential activation of distributed sensory-motor brain areas for different semantic word categories.

These studies do not, however, address the ambiguity of fMRI data with respect to the processing stage at which effects occur. In principle, all these results may reflect post-semantic processes such as mental imagery. Only few fMRI studies have addressed this issue directly. Tomasino et al. (2007) measured fMRI signals when participants read short phrases that were either action-related or not. They had to perform an imagery task, in which they were explicitly told to imagine the situation described by the phrase, or they had to perform a letter detection task. A difference in brain activation between action-related and non-action-related sentences occurred only in the imagery, but not in the letter detection task. The authors conclude that previous motor cortex activation results for action-words may have been caused by mental imagery processes. However, a letter detection task may have prevented not only mental imagery processes to take place, but may have been too superficial to even engage semantic processes. The results are therefore also consistent with the view that letter detection does not evoke semantic processing at a level that involves sensor-motor systems. Willems et al. (2010a) reported that action-words in a lexical decision task produced activation patterns in motor areas that were non-overlapping with activation patterns in a mental imagery task. Although this demonstrates that motor areas may play different roles in imagery and semantics, it also implies that motor cortex activation in non-imagery tasks cannot be fully explained in terms of mental imagery.

Wheatley et al. (2005) used a priming paradigm and could show that activity in left inferior temporal and left ventral premotor cortex, which were differentially activated by words referring to animate and manipulable objects respectively, were sensitive to semantic priming. Because the stimulus-onset-asynchrony between prime and target was only 150 ms, they argued that these areas are involved in the automatic processing of object meaning. Hauk et al. (2008b) studied the effect of semantic category on the word frequency effect. A negative modulation of brain activity by word frequency was found for visually related words in left inferior temporal areas, and for action-related words in left middle/superior temporal cortex. Assuming that a negative correlation with word frequency indicates processing at a lexico-semantic level, this is evidence that differential effects for semantic word categories indeed occur at a semantic rather than imagery stage. However, such a frequency effect was not observed in motor cortex.

In conclusion, a number of fMRI studies have provided evidence for the differential activation of sensory-motor areas during word and sentence comprehension, but the evidence that this reflects semantic rather than imagery processes is indirect and still scarce.

#### EEG/MEG

Fast psychophysiological methods such as EEG/MEG are less ambiguous with respect to processing stages. Although the exact time-course of lexico-semantic processes is still an ongoing research issue (Sereno and Rayner, 2000; Grainger and Holcomb, 2009; Hauk et al., 2012), we can assume that brain activity well before the earliest button presses in a lexical or semantic decision task (i.e., before ∼400 ms) are not related to mental imagery. Several studies have reported effects of semantic variables on brain responses already around 200 ms (Pulvermüller et al., 1999; Hauk et al., 2006; Amsel, 2011). Although fewer studies exist compared to the fMRI domain, reviews of the evidence for early semantic effects have already been provided (Pulvermüller, 2005; Hauk et al., 2008b; Kiefer and Pulvermüller, 2011). For example, Pulvermüller et al. (2001) and Hauk and Pulvermüller (2004) reported differences between action-word types in the ERP around 200 ms. Using a method to estimate the possible neuronal generators of these effects, the latter study found a pattern of results consistent with somatotopy. Similarly, differences between words with and without acoustic semantic features occurred in ERPs around 200 ms, and in a parallel fMRI study activation to words with acoustic features overlapped with areas activated during listening to sounds (Kiefer et al., 2008). Around the same latency, Moscoso del Prado Martin et al. (2006) observed ERP differences between color- and form-related words. Source estimation revealed more activation for form-related words in frontal brain areas, while color-related words activated temporal cortex.

All studies reviewed in the previous paragraph used visual word presentation. Visual words are easier to control for physical and psycholinguistic variables than speech stimuli, since the latter are extended over time and acoustic features can be difficult to quantify. However, of particular interest are studies using “mismatch negativity” (MMN) paradigms, which allow studying brain responses to stimuli outside the focus of attention (Pulvermüller and Shtyrov, 2006). Even when participants were distracted by watching a silent movie, the brain responses measured by MEG to auditorily presented words around 200 ms differed depending on the action-word category (Pulvermüller et al., 2005b). Source estimation revealed that leg-related words producing more activity around the vertex (i.e., consistent with leg motor cortex), and hand/mouth-related words activating more lateral areas (consistent with hand/mouth motor cortex). In addition to the “earliness” of these effects, the fact that they occur outside the focus of attention has been taken as evidence that they occur at an automatic semantic level, and are not under strategic control.

There is convergence among these studies that differences between semantic word categories occur around 200 ms after stimulus onset^2^. However, there are clearly fewer studies than in the fMRI domain. What is more, the analysis of EEG and MEG data is less standardized, and more difficult to compare across studies, than for fMRI. The fact that several studies using very different methodology converge on the same conclusion should be taken as support for their conclusions. At the same time, it is difficult to integrate these results in a common coordinate frame.

#### Transcranial magnetic stimulation

The strongest conclusions could potentially be drawn from TMS studies, which can test the effect of temporal stimulation of specific brain areas on behavioral performance. Effects of TMS on language comprehension and production are well-established (Devlin and Watkins, 2006), but evidence for a causal involvement of sensory-motor areas in semantic processing in this area of research is surprisingly scarce. As we have pointed out before, studies using MEPs provide correlational rather than causal evidence, and we will here focus on studies that have studied the effect of motor cortex stimulation on language performance.

Pulvermüller et al. (2005a) investigated the effects of TMS pulses delivered at 150 ms after word presentation to hand and leg motor cortex on performance in a lexical decision task. Target words were either hand- or leg-related. The authors found an interaction of stimulation site and word type, i.e., responses to arm-related words were faster after hand motor cortex was stimulated, and faster to leg-related words after leg motor cortex stimulation. The fact that response facilitation rather than inhibition was observed was attributed to the fact that TMS pulses were delivered at relatively low intensities. Tomasino et al. (2008) studied effects of TMS on hand-action-verb processing at different stimulation latencies and in different tasks. Sub-threshold TMS pulses were applied to hand motor cortex and vertex, respectively, at different latencies between 150 and 750 ms after word presentation. In different tasks, participants had to indicate by button press whether they had finished reading (silent reading), judge whether the action involved a rotation of the hand (imagery), or whether the word occurred frequently in a newspaper (frequency judgment). The main result was a facilitatory effect of hand motor cortex stimulation at all stimulation latencies, but only in the imagery task. The authors therefore argued that motor cortex is only involved in action-verb processing when it involves simulation of the corresponding movement. However, the silent reading task did not require any lexical or semantic processing at all in order to initiate a response (and there are no correct or incorrect responses in this task). The other two tasks are quite unfamiliar tasks, which elicited response times of about 1200 ms, compared to about 600 ms in the Pulvermüller et al. (2005a) study. It is therefore possible that a lexical decision task is more sensitive to effects of motor cortex stimulation.

In conclusion, direct evidence from non-invasive studies for a causal link between motor cortex and language processing is still scarce. In particular, there is no evidence yet that sensory-motor cortex stimulation disrupts semantic processing. Evidence of this kind has only been provided by studies on clinical populations, such as Parkinson’s disease (Boulenger et al., 2007; Herrera et al., 2012), stroke patients (Neininger and Pulvermüller, 2001, 2003; Trumpp et al., 2012), and semantic dementia (Pulvermüller et al., 2010). Kemmerer et al. (2010) compared behavioral measures of action comprehension and lesion overlap in a large group of brain damaged patients. Lesions in several brain areas including precentral gyrus, possibly extending into hand-related motor areas, as well as ventral postcentral gyrus predicted performance in tasks such as word-picture matching or word comprehension for action-verbs. This is probably the strongest neuropsychological evidence so far that these areas contribute to action-verb processing. However, using a similar approach but with smaller sample size, Arevalo et al. (2012) did not find evidence for somatotopic effects of different action-word categories.

### Abstract words

#### Behavior

In the behavioral domain, the ACE mentioned above for concrete sentences has also been observed for abstract sentences (Glenberg et al., 2008): participants performed a hand movement faster when the direction of movement was congruent with the direction of information flow (e.g., reading to somebody or being read to) described in a preceding sentence, compared to when it was incongruent. Similar effects have been observed with metaphors (Santana and de Vega, 2011). To our knowledge, this type of evidence has so far not been provided for abstract words in isolation. Several fMRI studies have investigated the embodiment of abstract sentences. For example, Boulenger et al. (2009) reported somatotopic activation for idiomatic sentences (“he grasps the idea” and “she kicks the habit”). Two other studies failed to find such effects for abstract sentences, which may be due to stimulus selection or experimental design (Aziz-Zadeh et al., 2006; Raposo et al., 2009).

#### fMRI

A number of studies have investigated effects of general word concreteness or abstractness, and for example have found more activation for abstract compared to concrete words in left inferior frontal cortex (e.g., Fiebach and Friederici, 2004; Noppeney and Price, 2004; Sabsevitz et al., 2005). However, only few fMRI studies have investigated differences between different abstract word categories with respect to embodiment. Ruschemeyer et al. (2007) used abstract German words that contained concrete action-words as stems (e.g., “be-greifen,” which means “to comprehend” and contains the stem “grasp”), but they did not activate motor cortex. Moseley et al. (2011) hypothesized that the meaning of emotion-words is grounded in emotion-expressing actions, and that “neural circuits controlling facial expressions and bodily actions related to an emotion concept like ‘anger’ are tightly linked to our neural representation of the word denoting it.” In line with this hypothesis, they found stronger motor cortex activation to emotion-words compared to non-action-related words. This was still the case when the analysis was restricted to emotion-words that did not directly refer to actions (such as “frown”).

#### EEG/MEG

As for fMRI, several EEG/MEG studies have investigated general effects of concreteness. Holcomb et al. (1999) and Adorni and Proverbio (2012) found a modulation of the N400 component by concreteness in sentence context. Amsel (2011) reported effects of multiple semantic variables, including imageability, already around 200 ms. However, without information about the neuronal sources these results do not provide direct evidence that early brain responses to abstract words contain signs of embodiment. In an MEG version of their previous fMRI experiment, Boulenger et al. (2011) presented literal and idiomatic sentences and analyzed the time-course of brain responses after the critical word (e.g., “habit” in “she kicked the habit”). Literal and idiomatic sentences differed in their brain responses after about 200 ms. Interestingly, there was also evidence for somatotopic activation for arm- and leg-related idioms in this latency range. To our knowledge, this is the only study so far that has tested theories of embodiment for abstract concepts using EEG/MEG, and no data from single-word studies are available so far.

#### Transcranial magnetic stimulation

Evidence from TMS studies is also rare. Glenberg et al. (2008) measured TMS-induced MEPs in their behavioral study described above, and found that MEP amplitudes were greater in transfer sentences than no-transfer sentences, and that there was little difference between concrete and abstract sentences. As explained before, MEPs do not allow inferences about causality of motor cortex in language processing. Pobric et al. (2008) demonstrated using repetitive TMS that disruption of right posterior superior temporal sulcus impaired processing of novel compared to conventional metaphors, but the effects of sensory-motor stimulation were not studied. Similarly, the involvement of left ventrolateral prefrontal cortex in abstract word processing has been demonstrated in neuropsychological and TMS data (Hoffman et al., 2010), but again this does not demonstrate a link between abstract concepts and sensory-motor brain systems. We are not aware of any direct evidence from neuropsychology or TMS that has demonstrated this link for abstract semantics yet.

### Numbers

In the previous section, we noted that evidence for embodied abstract word semantics from neuroimaging and neuropsychology is scarce. Importantly, evidence for a causal link between sensory-motor systems and processing of abstract words does not exist yet. We therefore ask here whether this evidence exists in the domain of numerical cognition. Numbers are an interesting case because they have no direct referent in the real world, but can assume different meanings in many different contexts. It has been suggested that the concept of numbers is shaped by sensory-motor experience during development. Finger-counting habits might impact on number semantics based on the way children acquire knowledge about numbers by counting with their fingers (e.g., Butterworth, 1999). This learning process may also involve innate systems for magnitude processing (Dehaene et al., 2003). Supporters of the embodied view on numerical cognition have proposed that systematic sensory-motor associations during number acquisition remain part of our numerical knowledge in form of conceptual metaphors (Lakoff and Nunez, 2000). Some review articles have already discussed the neuroscientific literature on numerical cognition from an embodied perspective (Andres et al., 2008; Fischer, 2012). We here review the evidence in the context of embodied abstract semantics and in the light of our methodological considerations above.

#### Behavior

Several studies have reported interference effects between spatial and motor information and performance during number processing. In the spatial domain, it has been demonstrated numerous times that participants make associations between number magnitude and space, e.g., along a “mental number line” (e.g., Izard and Dehaene, 2008). An important source of evidence stems for the “SNARC” (spatial-numerical association of response codes) effect, which means that participants usually respond faster to small numbers using their left hand and faster to larger numbers using their right hand (Dehaene et al., 1993; Wood et al., 2008). Although the exact direction of this association may be flexible (e.g., Shaki and Fischer, 2012), it is evidence for a link between spatial representations and number magnitude processing. However, a recent study suggests that this effect may not reflect properties of the semantic representations of numbers, but rather how we organize them in working memory in specific tasks (van Dijck and Fias, 2011). In the motor domain, evidence has been provided for the impact of numerical tasks on finger-counting related movements (Di Luca et al., 2006), and for priming of number magnitude through canonical finger-counting postures (Di Luca and Pesenti, 2008). Badets and Pesenti (2010) reported a motor-to-semantic interaction, showing that observed closing grip postures slowed down the processing of large numbers. Furthermore, an influence of individual finger-counting habits on the SNARC effect has been found (Fischer, 2008).

#### fMRI

In the fMRI domain, a recent study used multi-voxel-pattern-analysis (MVPA) to test for brain areas that are sensitive to the congruency between visually presented numerical and spatial intervals (Koten et al., 2011). These areas were intraparietal sulcus, frontal eye fields, and supplementary motor areas. With respect to motor cortex, it has been reported that brain activation evoked by visually presented small numerals in the precentral gyrus was lateralized according to whether participants report to usually use their left or right hand to gesture small numbers, i.e., small numerals mainly activated left-lateral premotor cortical regions in “right-starters,” and right-lateral premotor cortical areas in “left-starters” (Tschentscher et al., 2011).

#### EEG/MEG

Only few studies have addressed questions about the functional locus of the SNARC effect using ERP methodology. Two studies have provided evidence that spatial-numerical associations occur at a response preparation stage rather than during semantic processing, e.g., using lateralized readiness potentials (Keus et al., 2005; Gevers et al., 2006). To our knowledge, there are no ERP/ERF studies on finger-counting related effects in number processing.

#### Transcranial magnetic stimulation

Several TMS studies have addressed the role of motor circuits and spatial-numerical associations in number processing. Some of them measured the modulations of MEPs during numerical tasks, and consequently do not provide causal evidence (Andres et al., 2007; Sato et al., 2007). A few studies have shown that stimulation of parietal areas, in particular around angular gyrus, reduces the effect of spatial-numerical associations, e.g., in a line-bisection task (Gobel et al., 2001; Cattaneo et al., 2009) or in a SNARC paradigm (Rusconi et al., 2007). The effect in the latter study was attributed to disruption of the link between numbers and visuo-spatial attention rather than to interference with core number representations. One TMS study provided evidence for a causal role of angular gyrus in both number processing and finger movements (Rusconi et al., 2005). However, to our knowledge no study has investigated the direct effect of motor cortex stimulation on number processing performance yet. For a more complete overview of this research area, see Sandrini and Rusconi (2009). Neuropsychological evidence for a functional overlap between the spatial and motor system and number processing is provided by the Gerstmann Syndrome (Gerstmann, 1924; PeBenito, 1987), resulting from damage of the left parietal lobule, which results in difficulties with number processing, orientation in space, control of actions, and representation of own body shapes (for review, see Butterworth, 1999).

### Arithmetic

#### Behavior

In analogy to number processing, several behavioral studies have shown that arithmetic fact retrieval have an “operational momentum,” e.g., addition problems are associated with movements to the right and subtraction problems with movements to the left (Pinhas and Fischer, 2008; Knops et al., 2009). There is also behavioral evidence that simple arithmetic operations can involve finger-numerical representations in adults (Badets et al., 2010; Klein et al., 2011). For example, in a response-effect compatibility paradigm, Badets et al. (2010) observed faster responses to simple addition problems when congruent finger-counting gestures were presented. However, a recent study suggested that implicit finger-counting knowledge only impacted on simple arithmetic problem solving when participants were requested to use counting strategies (Imbo et al., 2011). This challenges the relevance of finger-counting knowledge for adults’ simple arithmetic, considering that adults mostly use memory retrieval strategies instead of counting when solving simple arithmetic problems (LeFevre et al., 1996, 2006).

#### fMRI

A number of fMRI and PET studies have shown activation in parietal and motor areas of the brain during arithmetic processing (see Arsalidou and Taylor, 2011 for review). However, to what degree these activations reflect embodied processes has only been addressed by a small number of studies. The operational momentum effect has been demonstrated in an fMRI study using an MVPA approach (Knops et al., 2009). This study showed that brain activation patterns in the frontal eye field were similar for real eye movements to the left and right on the one hand, and addition and subtraction problems on the other. Several fMRI studies have suggested a special role for visual-spatial processes in addition, and for verbal processes in multiplication (Chochon et al., 1999; Zhou et al., 2007; Grabner et al., 2009). In the motor domain, Andres et al. (2012) found common activation for mental calculation and finger representations in an fMRI conjunction analysis in bilateral horizontal intraparietal sulcus (hIPS) and posterior superior parietal lobule (PSPL), but not in motor cortex.

#### EEG/MEG and TMS

Only a few ERP studies investigated differences between arithmetic operation types with respect to sensory-motor concepts, and found evidence for stronger involvement of visual-spatial processes in addition and for verbal processes in multiplication in early time windows of arithmetic fact and rule retrieval (Zhou et al., 2006, 2009). To our knowledge, there is no evidence from EEG/MEG studies using source estimation that could shed light on the time-course of activation in sensory-motor systems during arithmetic fact retrieval. Similarly, evidence for a causal involvement of sensory-motor systems from TMS studies is still missing. In line with neuropsychological evidence from the Gerstmann Syndrome, specific impairment of simple arithmetic processes has been observed in patients with parietal cortex damage (Dehaene and Cohen, 1997; Lemer et al., 2003). However, there is no such evidence for impairment arithmetic skills due to lesions in motor cortices.

In conclusion, there is some correlational evidence for a role of spatial-numerical and motor associations in numerical cognition. This evidence stems mostly from studies on number perception, and to a lesser degree from studies on arithmetic fact retrieval. However, time-course information or evidence for a causal link between sensory-motor systems and numerical processing is currently non-existent or scarce.

## Conclusion

We have reviewed the theoretical and methodological challenges that are faced by the neuroscientific investigation of embodied semantics. Although there are several theoretical approaches that plausibly accommodate a role of sensory-motor systems in semantic processing (Harnad, 1990; Barsalou, 1999; Pulvermüller, 2012), it remains a challenging empirical question to what degree cortical sensory-motor systems contribute to semantics in the fully developed brain. Among the different interpretations of the concept “embodiment” (Wilson, 2002), we focused on the role of neuronal sensory-motor systems in semantics (Meteyard et al., 2010). Going back to the mental imagery debate (Kosslyn, 1975; Anderson, 1978), we pointed out that any measurement of behavioral or brain responses will necessary reflect a conflation of representations and processes, i.e., a mix of how information is stored and how it is retrieved in a particular context. We then highlighted the major strengths and weaknesses of neuroimaging methods fMRI, EEG/MEG, TMS for the investigation of sensory-motor systems in semantics, which guided our review of the empirical literature on this topic. Our review mainly covered the literature on concrete and abstract word processing, as well as number processing and arithmetic fact retrieval as special instances of abstract semantics.

### Summary and interpretation of the existing evidence

Our review of the empirical literature revealed that the bulk of evidence for embodied word semantics stems from fMRI studies on concrete words, which have demonstrated that the perception of words leads to activation in cortical sensory-motor systems depending on their referents. Unfortunately, fMRI data are correlational, have very low temporal resolution, and are arguably the least conclusive with respect to the functional interpretation of these effects. There are much fewer studies on this issue in the EEG/MEG than in fMRI domain. Nevertheless, the existing studies seem to converge on a latency of about 200 ms after stimulus onset for the earliest differences in brain responses between semantic word categories.

The interpretation of these effects as evidence for embodiment crucially depends on the neuronal generators of these signals, namely whether they originate in sensory-motor areas of the brain. The spatial resolution of EEG/MEG measurements, as well as source estimates derived from them, is inherently limited (Molins et al., 2008; Hauk et al., 2011). However, the existing evidence is consistent with the view that the generators of the early effects are distributed according to their sensory-motor associations, e.g., somatotopically in the case of action-words.

Only one TMS study has provided evidence for a causal link between sensory-motor systems and semantics. There is some evidence from neuropsychological studies that damage to sensory-motor areas can affect semantic processing. We conclude that there is strong evidence, although yet no proof, that cortical sensory-motor systems subserve concrete semantics. However, some crucial evidence on the time-course of sensory-motor activation in word processing, and in particular on the causal effects of sensory-motor activation on language performance, is still scarce and inconsistent.

The evidence for embodied abstract semantics is clearly weaker than for concrete semantics. Evidence for behavioral interactions exists at the sentence level, but not for single-words. Several studies have investigated fMRI responses to abstract sentences, such as idioms, but the findings are inconsistent. This may be due to experimental paradigms, stimulus selection and analysis methods, but clearly further research is needed to reconcile these studies. Evidence about the time-course of embodied abstract semantics, or the causal relationship between sensory-motor systems and abstract semantic processing, is almost non-existent. Similarly, research on numerical cognition has provided some evidence that sensory-motor systems may be involved in the retrieval and processing of numbers and arithmetic facts. The few ERP studies on this topic suggest that spatial-numerical associations play a role at the level of response selection rather than semantics. Questions about the time-course and causality of these effects should be addressed in more detail by further research. The techniques employed in research on concrete words may provide some guidance in this area.

How can the existing evidence inform theories of embodied semantics? Several theories posit a role for sensory-motor systems in semantic processing for concrete words (Harnad, 1990; Barsalou, 1999; Pulvermüller, 2012). Mechanistic models have been developed that describe how these theories may be realized by neuronal networks (Wennekers et al., 2006; Garagnani et al., 2008). It is therefore likely that the findings for concrete words reviewed above reflect the “grounding of the tree of semantic relationships” as in Harnad’s framework, or the bottom level of “convergence zones” in Barsalou’s framework, or “distributed category-specific networks based on Hebbian associations learning” in Pulvermüller’s framework.

However, based on the existing evidence, it is difficult to define the functional role of sensory-motor systems in semantic processing more precisely. Harnad and Barsalou describe the idea of convergence zones at different levels of abstraction. This may be illustrated on the basis of an example provided by Wilson (2002): We may start learning about the meaning of numbers by gesturing small numbers with our fingers. At the beginning, we fully flex the corresponding fingers. When we get better at this, we just briefly twitch them. At some point, even this may not be necessary any more, but our motor cortex may still be activated, e.g., to aid our short term memory. But why stop there? At some later stage, activation may only occur in areas that are several synaptic relays removed from motor cortex, e.g., in parietal or frontal lobes. It may then depend on the particular problem we need to solve, or information we need to retrieve from the stimulus, which of these neuronal systems contributes to performance. Do we need information from sensory-motor systems in order to decide whether “tree” refers to a tool or not, or can the necessary information be retrieved from higher-level convergence zones (Harnad, 1990; Barsalou et al., 2003) or the semantic hub (Patterson et al., 2007; Pulvermüller et al., 2010)? Does this differ from the task of determining whether trees can be blue? Are sensory-motor systems necessary for every task that involves semantic processing?

In research on number processing, behavioral and ERP evidence suggests that effects of spatial-numerical associations (e.g., the mental number line) do not occur at the level of semantic representations, but rather during later strategic processes such as working memory or response selection (Gevers et al., 2006; van Dijck and Fias, 2011). In conclusion, novel experimental paradigms and analysis methods are required to define the role of sensory-motor systems in semantic processing in more detail.

### Future directions: Behavior and connectivity

Very few studies so far have demonstrated effects of activation in sensory-motor systems on task performance in word processing. It is still possible that word stimuli automatically activate distributed semantic networks, but whether these affect performance is not clear yet, and it may depend on the particular task. Future studies using correlational measures such as fMRI or EEG/MEG could use activation values in specific brain areas as predictors for behavioral measures such as reaction times. Novel methods of functional and effective connectivity analysis may shed some light on the connectivity between sensory-motor areas and possible convergence zones or hubs (Valdes-Sosa et al., 2011).

Assuming that sensory-motor systems play an essential role in semantics, it is still an open question as to how the activity in these distributed areas is coordinated or bound together. Some authors have pointed toward the anterior temporal lobe as the semantic hub (Rogers et al., 2004; Patterson et al., 2007; Pulvermüller et al., 2010), while others have attributed this function to the angular gyrus, or possibly multiple regions (Binder and Desai, 2011). Novel methods for connectivity analysis may clarify this issue. If connection strengths between a brain region and sensory-motor systems were found to be modulated by semantic category (e.g., action- vs. object-word) or by task context (e.g., lexical vs. semantic decision), this would provide strong evidence that this region indeed serves as a hub. Even stronger evidence would be provided if these connection strengths also predicted behavioral performance, e.g., in categorization or identification tasks, or in naming.

EEG/MEG can be particularly useful in this endeavor, not just because they can distinguish “early” from “late” processing stages, but also because they allow different types of connectivity analyses. It is not yet clear how (or in how many different ways) brain areas communicate with each other. A possible candidate are oscillations (e.g., Fries et al., 2007), and functional connectivity may be reflected in coherence or phase-coupling among brain areas within and across frequency bands (Schoffelen and Gross, 2009). In addition, effective connectivity can be assessed using measures of Granger causality or structural equation modeling (Kiebel et al., 2009; Valdes-Sosa et al., 2011). Effective connectivity measures even allow inferences about sources that are not reflected in the measured signal, such as subcortical generators (David et al., 2011). These developments will provide powerful tools to disentangle the distributed neuronal networks underlying semantic processing.

### Future directions: Flexibility and automaticity of semantic processing

Surprisingly few neuroscientific studies have systematically investigated the effects of task modulation on semantic word processing. If they did, it was mainly in order to distinguish imagery from semantics, rather than to analyze semantic processing in more detail. There is growing evidence that word recognition is flexible (Balota and Yap, 2006; Norris, 2006), and that semantic word processing is sensitive to task demands (Martens and Kiefer, 2009; van Dam et al., 2012). A detailed investigation of the spatio-temporal brain dynamics under different well-defined task demands is still lacking, and should be the focus of future research on embodied semantics. For example using the methodological approaches mentioned in the previous section, one could test how well activity in sensory-motor systems predicts behavioral performance in tasks that require different levels of semantic detail, ranging from “abstract or concrete?” to “does it involve handling with the index finger?”.

A few recent studies have already investigated the effect of task demands on action-word processing. In an fMRI study, van Dam et al. (2012) investigated brain activation to words that had to be judged either for color or for action attributes. Areas in the left parietal lobes activated more for action-words than for abstract words, but only during action-related judgments. This was interpreted as evidence for flexible and context-dependent semantic processing. From these data it is not yet clear whether task demands affect early retrieval of semantic information, or later stages of processing. This type of experiment, for example systematically varying depth of semantic processing or type of semantic judgment, should also be performed with EEG/MEG methodology.

Furthermore, it will be important to test whether sensory-motor activation in semantics reflects the activation of the same neuronal populations as for example in movement execution of object perception, or whether it reflects the activation of different neuronal populations in the same areas. This can potentially be addressed using priming or adaptation paradigms (Henson and Rugg, 2003; Wheatley et al., 2005; Gold et al., 2006). Recent studies have introduced motor priming paradigms to the investigation of embodiment (Glenberg et al., 2010). In a recent combined EEG/MEG study, arm- and leg-related words were presented shortly after participants initiated the experimental trial themselves by button press (Mollo et al., 2011). In different blocks, they either pressed the button by finger or by foot, respectively. The button remained pressed until a letter string appeared. If this string was a real word, participants released the button as quickly as possible. If it was a pseudoword, they kept the button pressed until the end of the trial. In the source space analysis, the authors found an effect of congruency between effector used for the button press (finger or foot), and word type (arm- or leg-related). This congruency effect occurred around 150 ms after the onset of the letter string, and not only in motor cortex, but also in a left posterior superior temporal area. Thus, pre-activation of a specific part of the motor cortex led to word-type-specific modulation of brain activity in a non-motor language area at a very early stage of processing. This suggests that motor areas related to finger or foot movements are essential parts of neuronal cell assemblies for action-related words. Future studies could apply this paradigm with different movement and word types, and under varying task demands.

A particularly interesting case are words with multiple meanings. Studies on single-word processing usually (often implicitly) assume that a word read in isolation activates its dominant meaning (e.g., that “kick” refers to hitting something with the foot, rather than to the feeling you get from riding a roller-coaster). This can only be studied in sentence context, which was not the focus of this review. It has been suggested that concepts are composed of parts that are context-dependent, and other parts that are context-independent (Barsalou, 1982). The spatio-temporal dynamics of polysemy may provide intriguing evidence for the flexibility of semantic processing.

Theories of embodied concrete semantics can, to some degree, be translated to abstract semantics as long as abstract concepts bear some relationship to concrete entities, by means of abstraction or metaphor (e.g., Lakoff and Nunez, 2000; Glenberg et al., 2008). This is clearly a fruitful field for future research. The question remains whether sensory-motor systems are also involved in “pure” abstract semantics. We are able to acquire concepts without sensory experience, e.g., by means of discourse and context (Bloom, 2001). Aspects of this process can be modeled by means of latent semantic analysis (Landauer and Dumais, 1997; Louwerse and Ventura, 2005). It will therefore be an important question for future empirical studies to what degree abstract semantic processing is driven by higher-level convergence zones, and to what degree lower-level sensory-motor systems are involved.

## General Conclusion

We have demonstrated that even in a relatively circumscribed research area such as concrete and abstract semantics for single words, it is difficult to define the specific function of sensory-motor areas. The empirical evidence is still inconsistent, and its functional interpretation limited. As some authors have pointed out previously (Wilson, 2002; Meteyard et al., 2010), different interpretations of embodiment exist. The right question to ask may not be “embodied or not?” but rather “embodied to what degree?” The possibility that sensory-motor systems may contribute more or less to different types of semantic processes has so far received little attention in the neuroscientific literature, although similar arguments have been presented in the debate about the role of visual representations in mental imagery (Pylyshyn, 2002).

Furthermore, one may ask whether the role of sensory-motor systems in semantics, or more generally in cognition, differs among individuals – are some individuals more embodied than others? There is evidence that experience with particular types of concepts, e.g., in sport, music, and dance, may shape the way we process actions and language (Calvo-Merino et al., 2006; Beilock et al., 2008; Hoenig et al., 2011). The investigation of these questions will be an exciting endeavor for future research. However, as we have shown for the simple case of single-word processing, a number of important experimental and methodological challenges need to be addressed before we can arrive at firm conclusions. While our methods have certainly become more complex over the last few decades, the brain has not become simpler. The major scientific challenge will be to formulate questions that we are able to answer.

## Conflict of Interest Statement

The authors declare that the research was conducted in the absence of any commercial or financial relationships that could be construed as a potential conflict of interest.

## Footnotes

^1^We would like to thank Richard Henson for drawing our attention to this debate.

^2^For auditory stimuli, the reference latency is often not stimulus onset, but the point in time at which crucial information become available, such as the word recognition point.
